# Virtuous and Vicious Cycles of Arm Use and Function Post-stroke

**DOI:** 10.3389/fneur.2022.804211

**Published:** 2022-03-29

**Authors:** Belen R. Ballester, Carolee Winstein, Nicolas Schweighofer

**Affiliations:** ^1^Synthetic, Perceptive, Emotive and Cognitive Systems Laboratory, Institute for Bioengineering in Catalonia, Barcelona, Spain; ^2^Division of Biokinesiology and Physical Therapy, University of Southern California, Los Angeles, CA, United States

**Keywords:** stroke, neurorehabilitation, learned non-use, computational neurorehabilitation, decision-making, compensatory movement, wearable sensors

## Abstract

Large doses of movement practice have been shown to restore upper extremities' motor function in a significant subset of individuals post-stroke. However, such large doses are both difficult to implement in the clinic and highly inefficient. In addition, an important reduction in upper extremity function and use is commonly seen following rehabilitation-induced gains, resulting in “rehabilitation in vain”. For those with mild to moderate sensorimotor impairment, the limited spontaneous use of the more affected limb during activities of daily living has been previously proposed to cause a decline of motor function, initiating a vicious cycle of recovery, in which non-use and poor performance reinforce each other. Here, we review computational, experimental, and clinical studies that support the view that if arm use is raised above an effective threshold, one enters a virtuous cycle in which arm use and function can reinforce each other via self-practice in the wild. If not, one enters a vicious cycle of declining arm use and function. In turn, and in line with best practice therapy recommendations, this virtuous/vicious cycle model advocates for a paradigm shift in neurorehabilitation whereby rehabilitation be embedded in activities of daily living such that self-practice with the aid of wearable technology that reminds and motivates can enhance paretic limb use of those who possess adequate residual sensorimotor capacity. Altogether, this model points to a user-centered approach to recovery post-stroke that is tailored to the participant's level of arm use and designed to motivate and engage in self-practice through progressive success in accomplishing meaningful activities in the wild.

## Introduction

Current rehabilitation of upper extremities (UEs) in clinical settings often fails to improve the quality of life of people who have had a stroke for two main reasons. First, whereas principle-based (1) rehabilitation focused on improving UE function or on reducing impairment requires very large doses of intensive movement practice (2–5), such doses are far from being the norm in clinical settings, at least in the US (6) and in Europe (7). Second, rehabilitation is often “in vain”, as an important reduction in function and use is commonly seen subsequent to rehabilitation-induced gains. For example, it has been shown that patients experience functional deterioration during the 4 years following hospital discharge, which puts them back to where they were just 2 months post-stroke (8). Similarly, in a re-analysis of UE use of the immediate treatment group of the EXCITE trial, one-quarter of the participants showed a marked decrease in use in the 2 years following treatment (9).

**Figure 1:**
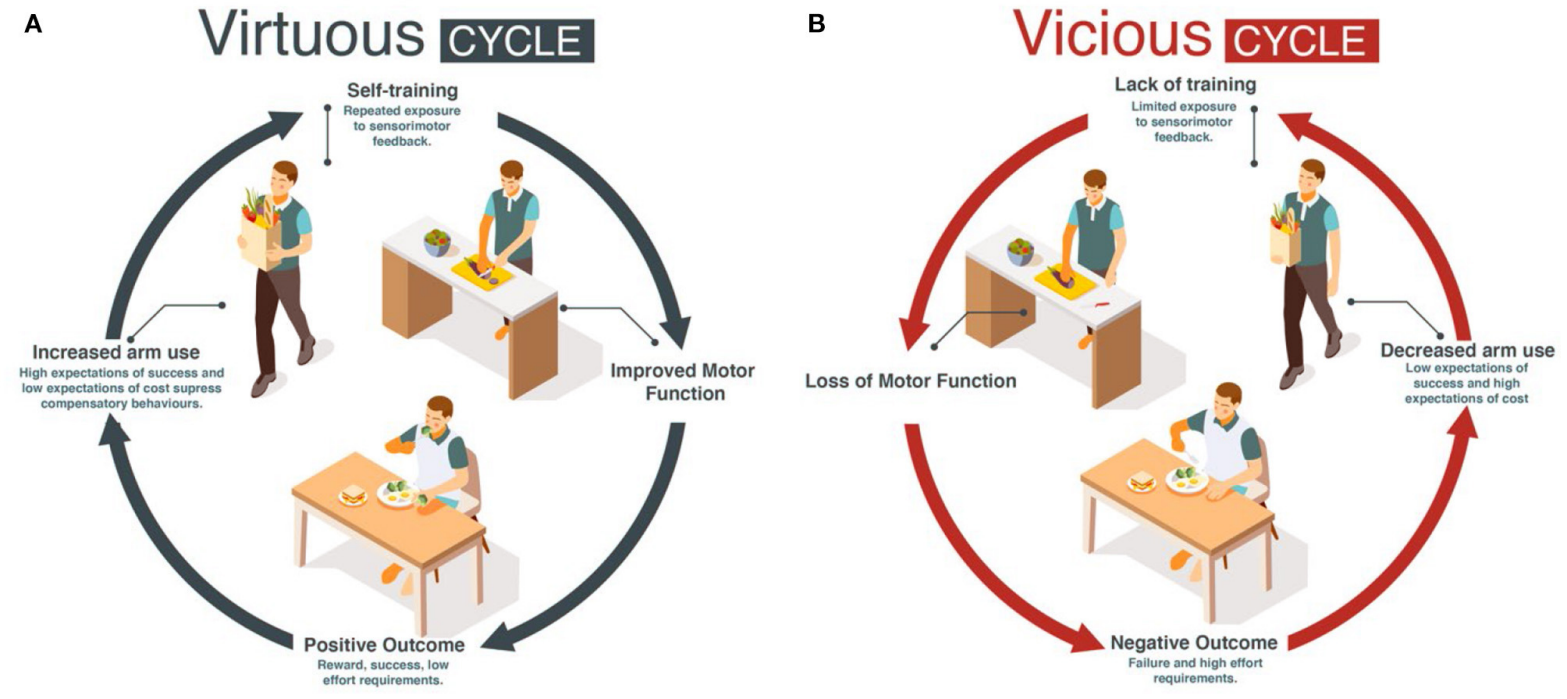
Conceptual model: the virtuous **(A)** and vicious **(B)** recovery cycles.

Here, we propose for mild to moderately impaired stroke survivors that increasing daily use of the more affected UE following motor therapy can solve the two above problems: if goal-directed UE movements in daily activities are seen as a single practice movement, such “self-practice” could potentially provide the large dose of movements and sensorimotor feedback needed to improve performance. The improvement in performance could then counteract the deteriorations in use by engaging the patients in a virtuous cycle of recovery, in which high levels of use and function reinforce each other (10) (Figure 1A). In contrast, a low level of use below a threshold may initiate a vicious cycle in which non-use and poor performance reinforce each other (Figure 1B) (10–12), leading to progressive deterioration in motor function and a further reduction in use.

In the following, we review earlier work that has led to the concept of virtuous and vicious cycles and more recent mechanistic models that yield such cycles. We then review experimental and clinical studies supporting such a “use it and improve it or lose it” phenomenon and the existence of an effective threshold in use and function separating the virtuous and vicious cycles. We next review recent work that aims at enhancing UE use in daily activities, including our recent work using ecological momentary assessment (EMA), and propose how to further develop the efficacy of embedding rehabilitation in the wild for those who possess enough residual sensorimotor capacity to benefit from more practice with motivational reminders to use the paretic limb.

## Acquired Non-use and the Origin of the Virtuous/Vicious Cycles of Recovery Post-stroke

**Figure 2:**
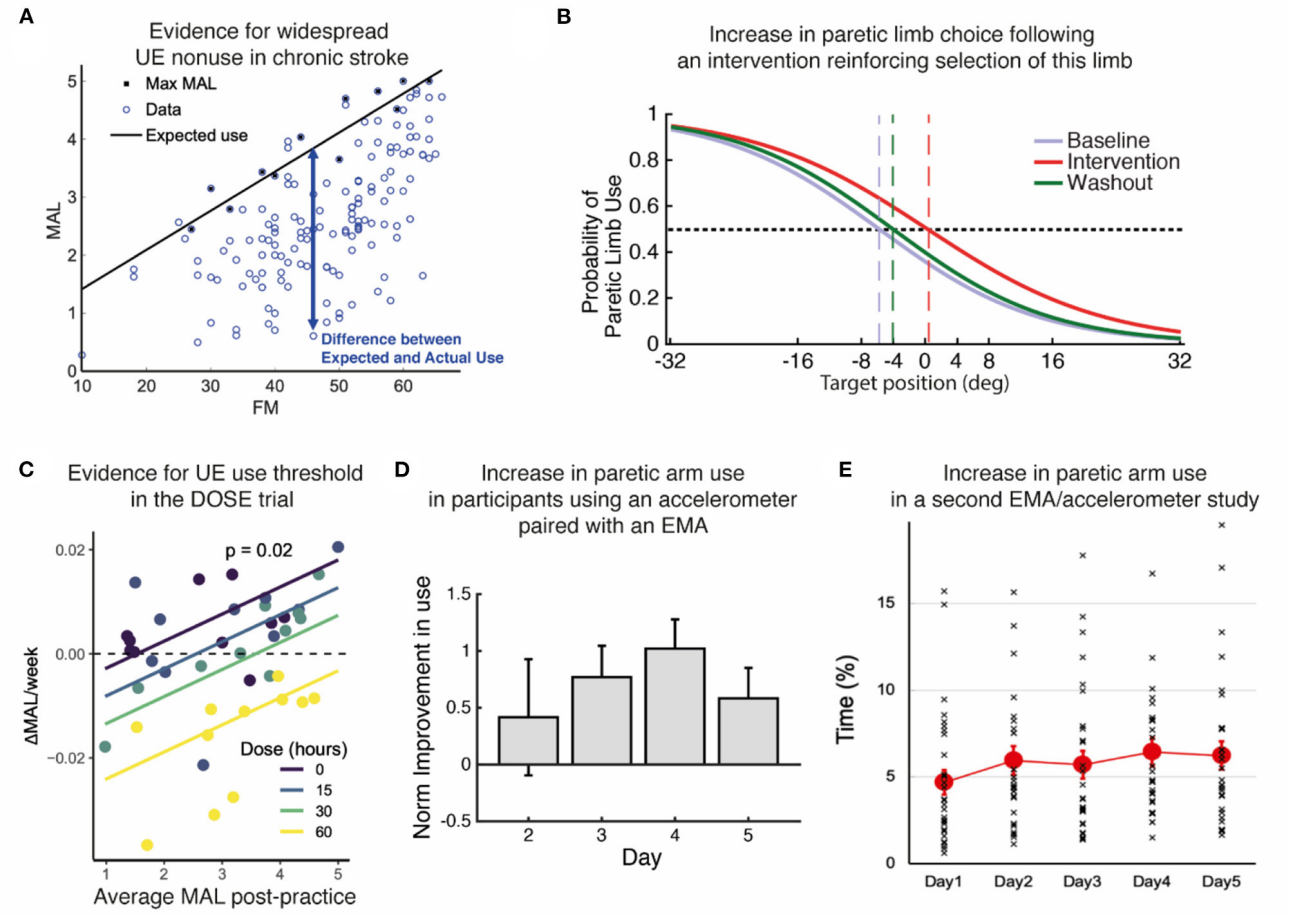
Summary of evidence. **(A)** Re-analysis of baseline data from the EXCITE trial (24) shows prevalent UE non-use beyond what is expected from impairment levels. Motor Activity Log Amount of Use sub-scale (MAL) as a function of the Upper Extremity Fugl Meyer (FM). We estimated the maximal use *MaxMAL* given impairment by the maximum MAL in each bin of 2 FM points (black line: *MaxMAL* = 0.067 × *FM* + 0.74). **(B)** Increase in paretic arm choice following an intervention that focuses on reinforcing the selection of this limb (25). Logistic fit of all subject's probabilities of paretic limb choice against target direction before (baseline), during, and after (washout) the intervention. Vertical dashed lines indicate targets with an equal probability of being reached with either arm. **(C)** Evidence for a threshold following practice in the DOSE study (26). The average weekly change in Motor Activity Log-Quality of Movement (MAL) following supervised practice is positively modulated by the average MAL post-practice for each dose. The intersections of the regression lines with the horizontal dashed line show the thresholds for each dose. Colored lines: retention rates as a function of average post-practice MAL for different doses; dots: individual retention rates. **(D)** Increase in paretic arm use in our first study using an accelerometer-embedded bracelet device paired with an EMA (25). Mean change in the activity of the paretic limb estimated by the wearable system across participants with respect to day 1 across days of intervention in which patients received haptic feedback and arm activity reports (days 2–4), and immediately after (day 5). **(E)** Small but statistically significant increase in paretic arm use over 5 days in our second study with an EMA / accelerometer combination (27). Solid color circles: daily average duration of unimanual right (paretic) arm/hand movements. Error bars, standard errors. Cross (×): individual use duration as a % of accelerometer wearing time.

The human motor system is highly redundant, offering multiple possible behavioral solutions to achieve a goal. When the behavior deviates from that observed in neurotypical individuals, we refer to the behavior as “compensatory” (13). For instance, individuals post-stroke frequently use their less affected hand to perform reaching movements. Because of the often large motor and sensory deficits immediately following stroke, the initial compensation is “mandatory”. However, as performance improves due to spontaneous recovery or rehabilitation, or both, the movements with the more affected arm are now possible but often not performed spontaneously (14, 15). Such “a non-use”, which is measured by the difference between what the patient can do when instructed and what the patient does when given a choice (16–18), was originally described as a learning process according to which preference for the less affected arm due to past unsuccessful repeated attempts to use the more affected UE (12, 19).^1^ It was also proposed that acquired non-use subsequently causes a loss of motor function leading to the acquisition of compensatory behaviors (12, 19, 21). Note that besides the loss of function, there may be other factors originating and perpetuating these compensatory behaviors, such as higher effort requirements associated with using the more affected limb (22, 23) and sensory perception and attention deficits after stroke. A re-analysis of baseline data from the EXCITE trial (24) shown in Figure 2A illustrates the often-considerable extent (and variability) of such acquired non-use in the chronic stroke population.

Acquired non-use has long been recognized to be a major issue facing patients post-stroke, but only recently has there been an attempt to operationalize this complex phenomenon where multiple physiological (e.g., sensorimotor impairment, lesion load), behavioral (e.g., expected success and effort), and psychological factors (e.g., self-efficacy, perceived effort) intersect (20, 28, 29). Although little is known about how these multiple factors interact to create acquired non-use, interventions that aim at reducing non-use have been developed. In particular, constraint-induced movement therapy (CIMT), which forces the use of the more affected by restraining the less affected limb with a mitt, while intensively training the use of the more affected arm, has been shown to reduce non-use (11, 24, 30, 31). However, CIMT is indicated for only ~15% of all stroke cases (e.g., being able to voluntarily extend the wrist and at least two fingers through a specified range of motion) (32). In addition, the mitt can only be worn by a few patients, requires supervision, and is often disliked. Importantly, the mitt prevents bimanual tasks, which make up the majority of daily arm and hand activities (33, 34). Furthermore, the common factor in CIMT and its variants is the delivery of a high number of repetitions. Besides compromising treatment adherence (35), large doses of practice are difficult to implement in the clinic, and, as we recently showed, highly inefficient (i.e., with low gain in outcome per hour of practice) (26).

## Virtuous and Vicious Cycles Arise From Plastic Interactions Between Motor and Decision-Making Systems: Insights From a Computational Model

Inspired by Taub's ideas, Han et al. proposed a computational model of brain plasticity and motor learning to unmask non-linear interactions between arm use and functional motor recovery (10). The model contains two main plastic neural processes: (1) A bilateral model of the motor cortex/cerebellum networks that generate reaching movements and that is (unilaterally) lesioned by stroke, and (2) a decision-making process, loosely based on the basal ganglia, that selects the arm to reach a given target. Motor performance is updated via plastic processes in the motor networks that reduce both errors and variability in movements. Motor decisions to choose one arm or the other depend on the between-arm comparison of expected future rewards, or “action values”, which are updated via plastic processes that aim at reducing reward prediction errors. Such choice mechanism is in line with studies showing that reinforcement modulates hand selection (36) and that effort plays a role in motor decisions (37–40). We confirmed since that arm selection depends on a context-dependent linear combination of the expected success and the anticipated cost for both arms in both neuro-typical (23) and post-stroke (20) individuals.

The Han et al. model predicted that if stroke suddenly decreases motor performance, the value of the more affected UE is down-regulated because of reach failures, leading to acquired non-use and compensatory choice of the less affected arm (10). In addition, the model predicted that recovery is bistable: following treatment, performance is either improving (recovery) or deteriorating. (Simulated) patients who use the affected arm above a threshold experience improved performance via “self-practice”. In turn, the amelioration of impaired performance increases use. The patients thus enter a chain of events in which improved performance leads to increase in use, which further improves performance and so on, resulting in a continuous process of improvement. Such a chain of events is called a “virtuous cycle” (Merriam Webster dictionary, 2022; Figure 1A). In contrast, (Simulated) patients who do not use the affected arm above this threshold enter a chain of events in which deteriorated performance leads to decrease in use, which further deteriorates performance, and so on, resulting in a continuous process of deterioration. Such a chain of events is called a “vicious cycle” (Merriam Webster dictionary, 2022; Figure 1B), with rehabilitation becoming “in vain”.

## Recent Evidence for the Virtuous and Vicious Cycles and the Threshold

Determining the threshold in use and function separating the virtuous and vicious cycles would allow for evidence-based decision-making of treatment schedules, preventing “rehabilitation in vain” and improving clinical outcomes. Recent work has been pursued toward the determination of such a threshold at the group level. In an animal study with rats who received focal ischemia, MacLellan et al., following the threshold hypothesis of the Han et al. model, theorized that functional benefit occurs only if a threshold of rehabilitation intensity is achieved (41). Skilled reaching improved in rats with unlimited access to the reaching apparatus in the dark but not when reaching was restricted. In addition, an enriched environment did not benefit the restricted group. This study showed that a critical threshold of rehabilitation intensity was required to obtain functional benefit.

Following the threshold prediction in the Han et al. model, we (42) performed a retrospective analysis of data from the EXCITE trial (24). We compared use of the paretic UE (assessed via the subjective Motor Activity Log Amount of Use scale) 1 week after therapy to use a year later. The paretic UE function (assessed via the Wolf Motor Function Test Functional Ability Scale-FAS) measured immediately after therapy predicted, on average, long-term changes of arm use: for about two-thirds of participants with function above a threshold (3.5/5.0 FAS), use improved. Below this threshold, use decreased. In a second re-analysis of the EXCITE trial data, this time using all repeated measures of UE use (assessed via the Motor Activity Log Amount of Use Scale) and function in the 2 years following treatment, we studied putative non-linear interactions between UE function and use (9). For this purpose, we largely simplified the Han et al. model via first-order non-linear dynamical models of change in use and function, which were fitted to the EXCITE data using a Bayesian regression framework. A model with reciprocal interactions between arm function and use was the best fitting model and accounted for the virtuous and vicious cycles. Furthermore, we found that therapy increased the parameter that modulated the effect of UE function on use. Simulations showed that increasing this parameter, which can be thought of as the confidence to use the arm for a given level of function (i.e., self-efficacy for paretic limb use), led to an increase in spontaneous use and the development of a virtuous cycle by decreasing the threshold.

In the recent DOSE clinical trial (4), in which participants were randomized into groups that varied in the duration of scheduled therapy (i.e., 0, 15, 30, or 60 h), we observed a dose response for the Motor Activity Log-Quality of Movement. In a later analysis of UE use in the 6 months following training (26), we modeled the change in use during and following task practice. Analysis of the model's retention rates in terms of UE use (i.e., Motor Activity Log Quality of Movement scale) demonstrated that when use was relatively high and above a threshold, it kept increasing. Interestingly, such an effect was more pronounced in the lower dosage groups because retention following task practice was worse in the higher dosage groups (see Figure 2C).

Whereas, the above studies from our group tested the threshold hypothesis with self-reported UE use at home, a recent study with stroke survivors in the subacute stage (43) tested the threshold hypothesis using data from a wearable device, the *Manumeter*, which has been shown to measure the amount of arm and hand movements (44). As predicted, spontaneous paretic hand use measured at home did not increase until the participants reached a certain level of function captured by a standardized clinical scale of motor dexterity (i.e., Box and Blocks Test). Recently, Chen et al. (27) monitored in the wild paretic limb use both alone (unimanual) and with the less affected limb (bimanual) over 5 days in a group of chronic stroke survivors with a wide range of FM motor scores (i.e., 20–66) and found in a subsequent analysis (45) that it was only in 16 out of 30 participants who had FM score > 50 that average unimanual paretic use time (% accelerometer wearing time) increased from 5 to ~15% as FM score increased to 66. In contrast, bimanual arm use linearly increased from 10 to 40% as FM score increased from 20 to 66. Thus, the paretic arm is being used to a greater extent in those with FM < 50, if it is embedded in the context of bimanual activities of daily living than when it is required to function alone. This suggests that there is a different impairment threshold for bimanual upper extremity activity that is much lower than that for unimanual paretic limb use. It also justifies our initial premise that those who are mild to moderately impaired (not exclusively those with mild impairment) are the ones who stand to benefit from this approach.

## Toward a Paradigm Shift: Embedding Rehabilitation in Activities of Daily Living With the Aid of Wearable Technology That Enhances Paretic Limb Use

The Han et al. model and the supporting evidence reviewed above suggest that increasing daily use (unimanual and bimanual) of the more affected UE following motor therapy can offer a solution to the problem of low dose of therapy observed in clinical settings and that of rehabilitation in vain. Because neurotypical and adults post-stroke make thousands of purposeful UE movements in daily activities (46), and such movements, via the feedback provided by the environment, could each be seen as single practice movements, increasing such self-practice could provide the large dose of movements needed to improve performance and then use.

In line with this vision, we explored a new method to promote arm use in stroke patients by boosting their confidence in more affected UE function. Participants with hemiparesis were exposed to reduced errors while performing arm shooting movements in a non-immersive virtual-reality system (25). Unaware of the manipulations, participants reported making internal attributions of the success they experienced through training and showed a higher probability of using their more affected arm (Figure 2B). We obtained similar results in a pilot study evaluating the effect of using an accelerometer-embedded bracelet device paired with ecological momentary assessment (“EMA” delivered on a smartphone) to monitor the amount of arm use and provide knowledge of progress in chronic stroke survivors (47). Participants received hourly haptic feedback and visual activity reports indicating the change from baseline in paretic arm use. The results showed a general increase in use of the more affected arm, and this increase was retained after feedback suppression (Figure 2D). Recently, we showed the feasibility of such EMA and sensor combination with thirty mild-severely motor-impaired stroke survivors (27). We found that the simple act of probing about arm use produced a small but significant increase (~10 min) in paretic arm use over 5 days (Figure 2E). In the same study, an analysis of EMA responses along with the quantitative accelerometer data revealed that social context (i.e., not alone) and self-efficacy for paretic arm/hand use complement an individual's motor capability (i.e., FM score) and play essential roles in paretic arm/hand use behavior in the natural environment (45, 48). Previous work from one of us showed that including social interaction in stroke VR-based motor rehabilitation enhances performance (49). Altogether, these studies illustrate the importance of social context, confidence, and reinforcement in restoring non-pathological hand selection patterns in stroke survivors. Future studies should shed light on which specific disability profiles would benefit the most from this type of intervention, as well as investigate whether this improvement in spontaneous use transfers to the participant's activities and increases independence.

For maximum effectiveness, the above studies suggest a personalized schedule of practice based on an individualized determination of use thresholds (unimanual and bimanual) via wearable sensors. When the different thresholds are estimated to be reached, supervised practice via EMA could be phased out, as UE use for daily activities would continue to increase. For individuals with UE use below these thresholds, strategies to overcome barriers to use in the natural environment would be needed to foster more effective engagement in self-practice (e.g., beginning with primarily bimanual tasks and then transitioning to more difficult unimanual paretic limb tasks). Thus, the next step is to determine this threshold for each individual patient. Repeated measurement of UE use in daily activities via sensors between bouts of therapy could yield precise threshold information. However, a difficulty is to monitor (via the sensors) and promote (via EMA) daily tasks that are at the “just right challenge” to maximize plastic processes involved in motor recovery. One solution is to use EMA + sensors (27) in conjunction with EMI (ecological momentary intervention) to design the optimal intervention strategy (48). As clearly shown by an early monkey study (50), not all repetitions yield plastic changes in the motor system: only the precision grasps, which had to be learned, and not the power grasps, were associated with motor cortical map plasticity. Thus, intelligent EMAs with sophisticated sensors that can monitor both arm and hand movements (e.g., bimanual and unimanual), such as the *Manumeter*, would need to promote a set of challenging tasks to practice, with this set varying during recovery to maintain challenge. Methods to define challenging task sets based on impairment levels have been proposed, e.g., (51). Note that in this scheme, the neurorehabilitation clinicians play an important role, as they need to assess whether or not particular movements, task components, and whole tasks are assigned so that real-world practice is engaging and productive.

In conclusion, the computational, experimental, and clinical studies reviewed above point to a user-centered approach to recovery post-stroke: besides the more traditional role of neurorehabilitation in enhancing motor function, and in line with standard therapy recommendations, we suggest that embedding rehabilitation in activities of daily living could largely improve long-term outcomes of stroke survivors. The development and validation of wearable devices for objective longitudinal monitoring and promotion of paretic arm/hand use both unimanually and bimanually may be key for implementing this rehabilitation approach that supports large doses of self-practice.

## Data Availability Statement

The raw data supporting the conclusions of this article will be made available by the authors, without undue reservation.

## Ethics Statement

Figure 2A presents results from a re-analysis of data from the EXCITE trial [(24), JAMA]. The trial was approved by the respective institutional review boards of each participating site of the EXCITE trial. The patients/participants provided their written informed consent to participate in this study.

## Author Contributions

All authors listed have made a substantial, direct, and intellectual contribution to the work and approved it for publication.

## Funding

This work was supported by NIH grants R21NS120274 to NS, R01HD065438 to NS and CW, R41HD104296 to CW, and the RGS@home project from H2020-EU, EIT Health (ID:19277).

## Conflict of Interest

NS is a co-founder of Motion Scientific, Inc., a company that is developing rehabilitation technologies. The terms of this arrangement have been reviewed and approved by the University of Southern California, Los Angeles in accordance with its conflict of interest policies. CW is a member of the data safety and monitoring board for Enspire DBS Therapy, Inc (DBS is Deep Brain Stimulation) and receives an honorarium for her services. She is a member of the external advisory board for MicroTransponder, Inc. and receives payment for her consulting. CW is Editor of the 6th edition of Motor Control and Learning, published by Human Kinetics, Inc and receives royalty payments. CW is also an Editor for the 2nd Edition of Stroke Recovery and Rehabilitation, published by DemosMedical Publishers and receives royalty payments. The remaining author declares that the research was conducted in the absence of any commercial or financial relationships that could be construed as a potential conflict of interest.

## Publisher's Note

All claims expressed in this article are solely those of the authors and do not necessarily represent those of their affiliated organizations, or those of the publisher, the editors and the reviewers. Any product that may be evaluated in this article, or claim that may be made by its manufacturer, is not guaranteed or endorsed by the publisher.

## Footnotes

^1^The original term “learned non-use” (LNU) was developed from studies in a monkey deafferentation model that is not the same as a stroke model. In spite of these different models, Taub et al. perpetuated the use of the term in their human stroke model work. However, there is no consensus as to what extent the observed non-use post-stroke is acquired through operant conditioning of early attempts to use the paretic limb that failed/negative reinforcement, or due to other factors such as diminished attention from reduced sensory perception, etc. Therefore, instead of the term LNU, we use “acquired non-use” to acknowledge the discrepancy between the deafferentation and stroke models. In our empirical and modeling work (10, 17, 20), we have developed an objective measure of “acquired non-use” using a paradigm first introduced by Sterr et al. (16) where we take the difference in the tasks or targets attempted in a “free” (spontaneous) condition in which the participant can choose either arm to capture the target, to that in a “forced” condition in which the participant must use the paretic limb to capture the target. The degree to which the “forced” condition is greater than the “free” condition captures the magnitude of spontaneous non-use and is consistent with the Andrews and Stewart article (14), “He can, but does he.” Our objective measure highlights the action selection process in arm use that precedes the actual behavior and that is of great interest to rehabilitation scientists who seek to develop interventions to promote the selection of the paretic limb spontaneously in the natural environment.
